# Percentiles of serum uric acid and cardiometabolic abnormalities in obese Italian children and adolescents

**DOI:** 10.1186/s13052-016-0321-0

**Published:** 2017-01-03

**Authors:** Rosa Luciano, Blegina Shashaj, MariaRita Spreghini, Andrea Del Fattore, Carmela Rustico, Rita Wietrzykowska Sforza, Giuseppe Stefano Morino, Bruno Dallapiccola, Melania Manco

**Affiliations:** 1 Department of Laboratory Medicine, Bambino Gesù Children’s Hospital, Rome, Italy; 2 Scientific Directorate, Bambino Gesù Children’s Hospital, Rome, Italy; 3 Nutrition Unit, Bambino Gesù Children’s Hospital, Rome, Italy; 4 Scientific Directorate, Research Unit for Multifactorial Disease, Bambino Gesù Children’s Hospital, Rome, Italy

**Keywords:** Cardiovascular disease, Childhood obesity, Insulin resistance, Metabolic syndrome, Uric acid

## Abstract

**Background:**

To investigate the association of serum uric acid (SUA) with cardiometabolic abnormalities in Caucasian overweight/obese children (<10 years of age) versus adolescents (≥10 years of age) by drawing age and gender specific percentiles of uric acid.

**Methods:**

Cross-sectional evaluation of 1364 Caucasian overweight/obese patients (age 4.1–17.9 years; 726 males, 53%; 560 children, 41%).

**Results:**

SUA levels were significantly lower in children than in adolescents (4.74 ± 1.05 vs. 5.52 ± 1.49 mg/dl, *p* < 0.001) and peaked in 12–14 years-old boys and 10–12 years-old girls.

In children with levels of SUA in the highest quartile (*N* = 75, 13%), OR for high triglycerides was 4.145, 95% CI 1.506–11.407 (*p* = 0.009). In adolescents with SUA in the highest quartile (*N* = 274, 34%), ORs for insulin resistance was 2.399 (95%CI 1.4–4.113; *p* < 0.001); for impaired fasting glucose 2.184 (95% CI 0.877–5.441; *p* = 0.07); for impaired glucose tolerance 2.390 (95% CI 1.405–4.063; *p* = 0.001); and for high triglycerides 1.8, (95%CI 0.950–3.420; *p* = 0.05). Multivariable random-effect linear regression models demonstrated that waist circumference and age (*p* < 0.0001 for both) are the variables most significantly predicting SUA levels, followed by triglycerides (*p* = 0.005) and 2 h glucose (*p* = 0.03) while HOMA-IR and BMI z-score did not predict SUA.

**Conclusions:**

High uric acid is associated with metabolic abnormalities and particularly with waist circumference very early in childhood.

## Background

Uric acid is the end-product of dietary and endogenous purine metabolism and results from the balance between hepatic production and renal excretion [1]. Serum uric acid (SUA) is an independent risk factor for atherosclerosis and cardiovascular disease (CVD) in adults [1–3] being associated in prospective studies with risk of developing metabolic syndrome (MetS), type 2 diabetes (T2D) and incident cardiovascular events [4].

In childhood and adolescence, SUA levels increase progressively from early childhood with body growth and plateau by ~15–17 years of age [5]. Concentrations in overweight and obese individuals are higher than in normal-weight peers [6, 7] and are associated with insulin resistance (IR) [8], cardiometabolic abnormalities belonging to the MetS [7–9], greater waist circumference (WC) [10], incident hypertension [11], increased carotid intima-media thickness [12] and impaired flow mediated dilation [13].

Recent studies focused on the association between SUA and cardiometabolic risk in young obese patients of different age-groups including prepubertal children [13–15].

The current study aims at investigating the association of serum uric acid with insulin resistance, disturbed carbohydrate metabolism, dyslipidemia and waist circumference in obese Caucasian children as compared to adolescents by providing age and sex specific percentiles of uric acid in young obese Italians.

## Methods

### Study population

The study population includes 1364 Caucasian overweight/obese children and adolescents (age range 4.18–17.93 years), referred by general practitioners to the Unit of Clinical Nutrition at the Bambino Gesù Children’s Hospital between July 2012 and 2013 [16]. Patients underwent anthropometric measurements, laboratory evaluation of uric acid, fasting glucose and insulin, lipid profile, liver function tests, white blood cell count (WBC); and a standard oral glucose tolerance test (OGTT). No child had genetic, renal or endocrine diseases, chronic illness, consumption of drugs affecting growth and carbohydrate metabolism, family history of symptomatic hyperuricemia.

### Anthropometric evaluation

Weight was measured with scales certified for medical use (90/384/EEC, SECA) with a precision of 50 g with children wearing minimal clothing and weight recorded to the nearest 100 g. Height was measured with a Holtain stadiometer and recorded to the nearest 0.5 cm. The average of two measurements was used. Children were classified as overweight/obese if BMI was ≥85th percentile according to Italian references [17]. WC was measured midway between the superior border of the iliac crest and the lower margin of the ribs at the end of normal expiration.

### Biochemical assays and estimation of IR

All the participants were asked to refrain from intensive physical activity in the 3 days prior to the study and were prescribed a standardized diet. Fasting blood samples were drawn after 8–12 h fast. Glucose and insulin levels were measured every 30 min starting from baseline up to 120 min following the OGTT (1.75 g of glucose/kg body weight up to a maximum of 75 g). The HOmeostasis Model Assessment of IR index (HOMA-IR) was calculated as average on two blood samples (-5 and 0 min) as [fasting glucose (mg/dl) x fasting insulin (μU/ml)/405]. Insulin sensitivity index (ISI) was calculated as [ISI  =  10,000/√(fasting glucose × fasting insulin) × (mean glucose × mean insulin).

SUA, glucose, total cholesterol, high-density lipoprotein (HDL) cholesterol, triglycerides, alanine aminotransferase (ALT) and gamma-glutamyl transferase (γ-GT) were measured by using commercial methods (ADVIA 1800 Chemistry System, Siemens Healthcare Diagnostic, Deerfield, IL). Serum insulin was analyzed by a chemiluminescent immunoassay method (ADVIA Centaur XP Immunoassay System; Siemens Healthcare Diagnostic, Deerfield, IL).

### Definition of metabolic abnormalities

Dyslipidemia was diagnosed in the presence of at least one of the following conditions: value of cholesterol and/or triglycerides higher than the 95^th^ percentile and/or HDL cholesterol lower than the 5^th^ for age and sex according to the American Academy of Pediatrics [18]. The triglycerides to HDL-cholesterol ratio >2.2 was considered atherogenic [19, 20]. Impaired fasting glucose (IFG) was defined as fasting glucose ≥100 mg/dl; impaired glucose tolerance (IGT) as 2 h glucose ≥140 mg/dl following the OGTT. Insulin resistance was defined as HOMA-IR value ≥75^th^ percentile adjusted for age and sex according to reference value of HOMA-IR in the Italian population as described elsewhere [21]. Hyperuricemia was defined as SUA value ≥75^th^ percentile adjusted for age and sex.

### Statistical analysis

Continuous variables were expressed as mean ± standard deviation (SD) and categorical variables as number and percentage. Normal distribution was tested using the Kolmogorov-Smirnov test. Between-group comparison was performed by using the χ^2^ test for categorical variables and ORs calculated. The Mann-Whitney U test was used for comparison of continuous variables. Uric acid distribution was tabulated for the values corresponding to the 5th, 10th, 25th, 50th, 75th, 90th, and 95th percentiles. Correlations were sought by using the Spearman test and variables significantly associated with SUA (age, BMI Z-score, waist circumference, triglycerides, HOMA-IR and 2 h plasma glucose entered a multivariable random-effect linear regression models to evaluate the association of SUA with metabolic abnormalities.

Statistical analyses were performed using the SPSS 21 statistical package (SPSS, Chicago, IL, USA). A result with *p* < 0.05 was considered statistically significant.

## Results

### Description of the sample

**Fig. 1:**
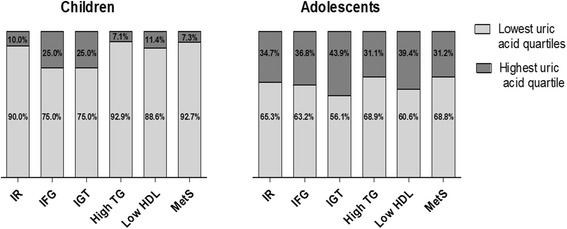
*Bars* represents 100% of cases with insulin resistance (IR), impaired fasting glucose (IFG), impaired glucose tolerance (IGT), high triglycerides (TG), low high densitity lipoprotein cholesterol (HDL), metabolic syndrome (MetS). *Dark grey* are cases with high uric acid above the highest quartile, *light grey* cases with uric acid in the lowest quartiles

Table 1 describes the study population as a whole, age and sex subgroups. A total of 1364 overweight/obese patients were studied, 560 (41%) children, 726 (53.2%) were males. Dyslipidemia was diagnosed in 366 (26.8%) patients; low HDL-cholesterol in 29 (2.2%); high total cholesterol in 252 (18.5%) and high triglycerides in 87 (6.4%). Children affected by dyslipidemia were 162 out of 560 (29%) and adolescents 204 out of 804 (25.4%). Disturbed carbohydrate metabolism was found in 94 patients, [25 children out of 560 (4.4%) and 69 out of 804 adolescents (8.6%)]: 41 with IFG (3%) and 53 with IGT (3.9%). IR was found in 209 patients (15.3%); 41 children (7.3%), 168 adolescents (20.9%). Table 2 reports SUA (mg/dl) in cases with metabolic abnormalities. Figure 1 show percentage of cases presenting with each metabolic abnormality and SUAlevels in the highest quartile of the distribution.

**Table 1:** Characteristics of the study population

	Total	Male	Female	*p value*		<10 years	≥10 years	*p value*
	1364	(*N* = 726)	(*N* = 638)			(*N* = 560)	(*N* = 804)	
Age (years)	10.74 ± 2.70	10.60 ± 2.48	10.83 ± 2.93	0.707	Male	290 (39.9%)	436 (60.1%)	
Weight (kg)	60.64 ± 18.74	61.19 ± 18.83	60.00 ± 18.63	0.433		45.98 ± 9.21	70.85 ± 16.82	<0.001
Height (cm)	147.22 ± 14.06	147.97 ± 14.28	146.36 ± 13.77	0.199		134.76 ± 9.31	155.89 ± 9.59	<0.001
Waist circumference (cm)	80.58 ± 10.21	81.95 ± 10.48	78.99 ± 9.65	<0.001		74.56 ± 7.62	84.90 ± 9.63	<0.001
BMI (kg/m^2^)	27.32 ± 4.36	27.28 ± 4.06	27.34 ± 4.67	0.811		25.13 ± 3.11	28.84 ± 4.45	<0.001
BMI z score (SDS)	2.03 ± 0.51	2.01 ± 0.50	2.06 ± 0.52	0.051		2.00 ± 0.39	2.05 ± 0.58	0.802
HOMA-IR	2.87 ± 1.98	2.75 ± 2.00	3.01 ± 1.94	0.001		2.43 ± 1.79	3.44 ± 1.94	<0.001
ISI	4.12 ± 2.50	4.23 ± 2.18	3.98 ± 2.82	<0.001		2.43 ± 1.79	3.50 ± 2.95	<0.001
Fasting serum glucose (mg/dl)	79.53 ± 10.03	80.38 ± 9.85	78.54 ± 10.15	0.001		78.06 ± 9.93	79.53 ± 10.03	<0.001
2 h serum glucose (mg/dl)	105.01 ± 18.79	105.41 ± 18.94	104.54 ± 18.59	0.367		102.07 ± 18.42	107.04 ± 18.77	<0.001
Fasting insulin (μUI/l)	14.40 ± 9.56	13.67 ± 10.03	15.23 ± 8.92	<0.001		11.57 ± 7.92	16.37 ± 10.10	<0.001
2 h insulin (μUI/l)	81.30 ± 68.17	72.28 ± 59.32	91.65 ± 75.82	<0.001		66.49 ± 63.68	91.50 ± 69.32	<0.001
Uric acid (mg/dl)	5.20 ± 1.38	5.28 ± 1.45	5.10 ± 1.30	0.058		4.74 ± 1.05	5.52 ± 1.49	<0.001
Total cholesterol (mg/dl)	156.46 ± 30.85	158.63 ± 32.82	154.00 ± 28.27	0.005		158.13 ± 28.67	155.30 ± 32.25	0.005
HDL-cholesterol (mg/dl)	48.86 ± 12.51	49.88 ± 13.78	47.70 ± 10.78	0.004		50.73 ± 12.89	47.56 ± 12.08	0.034
Triglycerides (mg/dl)	76.33 ± 41.02	76.84 ± 44.10	75.74 ± 37.01	0.382		71.39 ± 39.30	79.68 ± 41.86	<0.001
ALT (IU/l)	24.54 ± 19.18	26.86 ± 23.68	21.92 ± 11.73	<0.001		23.01 ± 10.74	25.61 ± 23.30	0.649
γ-GT (IU/l)	13.90 ± 8.98	15.02 ± 10.57	12.57 ± 6.41	<0.001		13.30 ± 5.98	14.30 ± 10.52	0.431

Data are expressed as mean ± SD or number and percentage. *BMI* body mass index, *HOMA-IR* homeostasis model assessment for insulin resistance, *ISI* insulin sensitivity index, *HDL-cholesterol* high-density lipoprotein cholesterol, *TG* triglycerides, *ALT* alanine aminotransferase, *γ-GT* gamma-glutamyl transferase

**Table 2:** Mean values of serum uric acid (mg/dl) in children and adolescents with and without metabolic abnormalities

Whole sample (*N* = 1364)		*p value*	Children (*N* = 560)		*p value*	Adolescents (*N* = 804)		*p value*
Normal Total cholesterol (*N* = 1112)	High Total cholesterol (*N* = 252)		Normal Total cholesterol (*N* = 452)	High Total cholesterol (*N* = 108)		Normal Total cholesterol (*N* = 660)	High Total cholesterol (*N* = 144)	
5.2 ± 1.4	5.2 ± 1.2	0.7	4.7 ± 1.0	4.8 ± 0.9	0.6	5.5 ± 1.5	5.5 ± 1.3	0.7
Normal triglycerides (*N* = 1277)	High triglycerides (*N* = 87)		Normal triglycerides (*N* = 518)	High triglycerides (*N* = 42)		Normal triglycerides (*N* = 759)	High triglycerides (*N* = 45)	
4.9 ± 1.4	5.3 ± 1.3	<0.003	4.4 ± 1.0	4.8 ± 1.0	0.03	5.3 ± 1.5	5.8 ± 1.3	0.9
Normal HDL-cholesterol (*N* = 1214)	Low HDL-cholesterol (*N* = 150)		Normal HDL-cholesterol (*N* = 516)	Low HDL-cholesterol (*N* = 44)		Normal HDL-cholesterol (*N* = 699)	Low HDL-cholesterol (*N* = 105)	
5.0 ± 1.4	5.5 ± 1.4	<0.001	4.6 ± 1.1	4.9 ± 0.9	0.03	5.3 ± 1.5	5.8 ± 1.5	0.02
Insulin sensitive (*N* = 1155)	Insulin resistant (*N* = 209)		Insulin sensitive (*N* = 519)	Insulin resistant (*N* = 41)		Insulin sensitive (*N* = 636)	Insulin resistant (*N* = 168)	
5.1 ± 1.3	5.7 ± 1.7	<0.001	4.7 ± 1.0	5 ± 1.3	0.5	5.5 ± 1.5	6 ± 1.7	0.5
NFG (*N* = 1323)	IFG (*N* = 41)		NFG (*N* = 547)	IFG (*N* = 13)		NFG (*N* = 776)	IFG (*N* = 28)	
5.2 ± 1.4	5.4 ± 1.3	0.4	4.7 ± 1.0	4.4 ± 0.9	0.4	5.5 ± 1.5	5.7 ± 1.3	0.9
NGT (*N* = 1311)	IGT (*N* = 53)		NGT (*N* = 548)	IGT (*N* = 12)		NGT (*N* = 763)	IGT (*N* = 41)	
5.1 ± 1.2	6.09 ± 1.4	<0.0001	4.7 ± 1.0	5.3 ± 1.1	0.05	5.4 ± 1.3	6.2 ± 1.5	0.002

Data are expressed as mean ± SD. *NFG* normal fasting glucose, *IFG* impaired fasting glucose, *NGT* normal glucose tolerance, *IG* impaired glucose tolerance

### Distribution of serum uric acid

Table 3 reports SUA percentiles from the 5th to 95th in the population grouped by sex and age. In boys, SUA rose significantly from >10–12 years to >12–14 years (*p* < 0.05) and from >12–14 years to >14 years (*p* < 0.05). In girls, SUA increased significantly from >8–10 years to >10–12 years (*p* < 0.05) when its levels peaked. Significant sex-related differences were found by 10 years of age. Boys aged 10–12 years had significantly lower SUA than age-matched girls (5.09 ± 1.30 vs. 5.41 ± 1.36; *p* = 0.002). Conversely, males had higher concentrations than girls at 12–14 years of age (6.02 ± 1.77 vs.5.17 ± 1.16; *p* < 0.001) and onward (6.99 ± 0.99 vs. 5.37 ± 1.56; *p* < 0.001).

**Table 3:** Distribution of serum uric acid (SUA, mg/dl) in overweight/obese patients by sex and age

			SUA percentile						
	Number	Age (years)	5th	10th	25th	50th	75th	90th	95th
Male									
	109	4–8	3.01	3.48	3.98	4.73	5.48	5.98	6.85
	178	>8–10	3.29	3.60	3.97	4.60	5.22	6.00	6.30
	229	>10–12	3.44	3.80	4.30	4.90	5.70	6.51	6.95
	143	>12–14	3.72	4.30	4.90	5.80	6.85	7.86	8.74
	67	>14	5.50	5.76	6.20	6.90	7.70	8.32	8.66
Female									
	104	4–8	3.16	3.29	4.01	4.67	5.22	6.36	6.81
	166	>8–10	3.10	3.56	4.10	4.70	5.50	6.10	6.67
	161	>10–12	3.80	4.10	4.60	5.30	6.05	6.70	7.19
	108	>12–14	3.34	3.60	4.30	5.10	5.87	6.80	7.00
	99	>14	3.28	3.89	4.40	5.15	6.02	7.11	7.50

High SUA (SUA ≥75^th^ percentile for age and sex) was observed in 246 patients (18%), 98 children out of 560 (17.5%) and 148 adolescents out of 804 (18.4%).

### Correlations

In the whole sample, there were statistically significant even weak correlations between SUA and age (ρ = 0.332), BMI (ρ = 0.416), BMI-z-score (ρ = 0.288), waist circumference (ρ = 0.467), HOMA-IR (ρ = 0.205); TG/HDL ratio (ρ = 0.218), fasting insulin (ρ = 0.238), ALT (ρ = 0.227) and ISI (ρ = -0.239) (*p* < 0.001 for all). SUA was also correlated with γ-GT (ρ = 0.167), triglycerides (ρ = 0.165), HDL (ρ = -0.149), fasting glucose (ρ = -0.107, *p* < 0.001) and ISI (ρ = -0.239) γ-GT (ρ = 0.167), triglycerides (ρ = 0.165), HDL (ρ = -0.149), fasting glucose (ρ = -0.107, (*p* < 0.001 for all).

Multivariable random-effect linear regression models demonstrated that waist circumference and age (*p* < 0.0001 for both) are the variables most significantly predicting SUA levels, followed by triglycerides (*p* = 0.005) and 2 h glucose (*p* = 0.03) while HOMA-IR and BMI z-score did not predict SUA (Table 4).

**Table 4:** Age and metabolic parameters predicting levels of serum uric acid

	Model 1	*p*	Model 2	*p*	Model 3	*p*	Model 4	*p*
Age			0.092 [0.053–0.130]	*<0.0001*	0.096 [0.057–0.134]	*<0.0001*	0.096 [0.058–0.134]	*<0.0001*
BMI z-score								
HOMA-IR								
2 h glucose							0.005 [0.001–0.010]	*0.03*
Triglycerides					0.003 [0.001–0.006]	*0.001*	0.003 [0.001–0.005]	*0.005*
Waist circumference	0.049 [0.041–0.058]	*<0.0001*	0.035 [0.025–0.046]	*<0.0001*	0.031 [0.020–0.041]	*<0.0001*	0.030 [0.020–0.041]	*<0.0001*

Values are regression coefficients and 95% confidence intervals (in brackets) obtained from multivariable random-effect linear regression

In children with levels of SUA in the highest quartile, ORs for high triglycerides and atherogenic ratio were 4.145, (95% CI 1.506–11.407; *p* = 0.009) and 2.74 (95%CI 1.001–7.343; *p* = 0.05), respectively. In adolescents, ORs were for insulin resistance 2.399 (95%CI 1.4–4.113; *p* < 0.001); IFG 2.184 (95%CI 0.877–5.441; *p* = 0.07); IGT 2.390, (95% CI 1.405–4.063; *p* = 0.001); high triglycerides 1.8 (95%CI 0.950–3.420, *p* = 0.05) and atherogenic ratio 2.354, (95%CI 1.490–3.718; *p* < 0.0001).

## Discussion

Our study reports first age-and sex-specific percentiles of serum uric acid in a very large population of overweight/obese young Caucasians of Italian ancestry. It confirms associations of serum uric acid with waist circumference, triglycerides and glucose tolerance (2 h glucose) in this population. Mean values of uric acid were significantly higher in children in the highest quartile of uric acid and both in children and adolescents with impaired glucose tolerance. Conversely, to belong to the highest quartile of uric acid was associated with an increased risk of presenting high triglycerides and atherogenic profile both in children and adolescents, while limited to adolescents there was an increased risk of presenting impaired fasting glucose and insulin resistance.

Gender-related trends of SUA concentrations over-time found in previous studies [5, 22, 23] were confirmed in our sample. In a nationally representative population of 6768 youths aged 12 to 17 years from the National Health And Nutrition Examination Survey, NHANES [5], concentrations of SUA raised in the peri-pubertal period, peaked at the puberty (i.e. 10–12 years of age in females and 12–14 in males) and reached adult levels soon afterward. Gender-related differences in the peaking time almost certainly reflects the different tempo of the puberty onset [18] with delayed sexual and skeletal maturation in males [5]. After the puberty and throughout the adolescence SUA levels are lower in females likely because of the uricosuric effect of estrogens on the kidney tubular reabsorption of UA [22] and higher in males as possible effect of androgens [23]. In the later adolescence, i.e. ages 15 to 17 years for males and 13 to 17 years for females, BMI, skinfold thickness and blood pressure were determinants of SUA concentration stronger than sexual and somatic maturation [5]. Indeed, in our series of obese patients, mean values were higher than those found in normal-weight individuals as reported by the NHANES [22].

The study of Tang et al. [7] investigated SUA concentration in obese children and adolescents of Japanese nationality. The authors observed such an association between SUA and MetS also in children that they concluded hyperuricemia should be yet considered in early childhood as CVD risk factor [7]. Very recently Bassols et al. [14] found increased serum uric acid is associated with cIMT in asymptomatic prepubertal children highlighting the role of uric acid levels in the shaping of the cardiometabolic risk very early in childhood.

Our findings are in keeping with these reports. SUA was correlated with metabolic abnormalities both in children and in adolescents. Patients in the highest quartile of SUA levels were heavier and presented with worst lipid and insulin metabolism. The best correlation was seen between levels of SUA and waist circumference. Ford et al. [10] explored the association between SUA and components of the MetS in a nationally representative sample of 1370 adolescents aged 12–17 years from the NHANES 1999–2002. Patients with the full MetS had the highest SUA concentrations. The strongest association of SUA was seen with the waist circumference. In the general population of NHANES, a concentration of SUA >5.7 mg/dL was associated with an adjusted OR for MetS of 14.79 (95%CI 7.78–28.11) [10]. In our population of exclusive obese patients, this cut-off value corresponded to the 90^th^ percentile of SUA in obese males aged 8 years-old; to the 75^th^ in those aged 10 years-old; and to the 50^th^ and 10^th^ in males aged 12 and older than 14 years of age, respectively. In girls, it corresponds to the 90^th^ percentile in girls 6 years-old and to the 50^th^–75^th^ in those by the age of 10 years onward. Hence, most of our obese patients had values of uric acids higher than 5.7 suggesting the co-occurrence of metabolic abnormalities. One of the limitations of the present study was the lack of Information on blood pressure. Therefore, we could not estimate prevalence of the full MetS and test the diagnostic accuracy of the 5.7 mg/dL cut-off as well as the association between SUA and blood pressure. It was a shame since uric acid contributes significantly to the pathogenesis of high blood pressure by activating the renin–angiotensin–aldosterone system directly at the vascular level and indirectly by inducing tubule-interstitial disease and consequent impaired baroreflex function [24]. High levels of uric acid in childhood were associated with high blood pressure at the same age [11]. The Bogalusa heat study demonstrated that high SUA in childhood predicts high blood pressure in adulthood [25] and, *viceversa,* features of MetS in childhood predict high SUA in adulthood [26].

Despite SUA is a CVD risk factor independent of IR [27], there is a clear relationship between SUA and hyperinsulinemia/IR levels. Hyperisulinemia/IR cause both increased production and reduced renal clearance of urate [28] while the amelioration of IR by low-energy diet or insulin sensitizing agents was found to decrease SUA levels [8]. Insulin favors renal urate reabsorption by stimulating the urate-anion exchanger URAT1 and/or the Na^+^-dependent anion cotransporter in brush border membranes of the renal proximal tubule [29]. In keeping with this pathogenic evidence, SUA levels paralleled insulin concentrations in our sample.

The strong association found by us and others [10, 13] with waist circumference confirms the strong link between uric acid, visceral adiposity and insulin resistance. In such interplay, increased consumption of dietary fructose may contribute [30] since fructose ingestion induces hepatic *de novo* lipogenesis, leading to increased visceral fat accumulation and, in turn, to worsened IR. One pathway to the production of serum uric acid, indeed, is via dietary fructose that activates the fructokinase metabolic system and upregulates *de novo* purine nucleotide synthesis in hepatocytes [31]. In that, SUA would act as marker of impaired adipogenesis [32] as further confirmed by associations observed between SUA and triglycerides levels, reduced HDL-cholesterol and, even better with an enhanced ratio of triglycerides to HDL-cholesterol after adjusting for covariates as seen in our population.

In addition to the lack of information about blood pressure, other important limitations must be acknowledged in our study, such as the absence of normal weight controls and information on pubertal stage.

In spite of these limitations, strengths of the present investigation were the large sample-size and the wide age-range that included preschoolers. Findings of relatively increased levels of uric acid already at this age supports the hypothesis of an early origin of cardiovascular disease associated with obesity putted forward by our research group [33].

## Conclusion

In conclusion, our study confirms in a large population of Italian overweight/obese children and adolescents the association of high serum uric acid and metabolic abnormalities belonging to the MetS. The potential role of SUA as marker of increased CVD relies on these associations and seems independent of the degree of insulin resistance.

There is need of further research in large cohorts of children and adolescents better phenotyped in relation to their CVD to prove the cost-effectiveness of measuring routinely and monitoring overtime SUA in clinical settings.

## Abbreviations

ALT: Alanine aminotransferase
AUC: Area under curve
BMI: Body mass index
CRP: C-Reactive protein
CVD: Cardiovascular disease
HDL-cholesterol: High-density lipoprotein cholesterol
HOMA-IR: Homeostasis model assessment for insulin resistance
IFG: Impaired fasting glucose
IGT: Impaired glucose tolerance
IR: Insulin resistance
ISI: Insulin sensitivity index
MetS: Metabolic syndrome
OGTT: Oral glucose tolerance test
SUA: Serum uric acid
T2D: Type 2 diabetes
TG: Triglycerides
WBC: White blood cell count
WC: Waist circumference
γ-GT: Gamma-glutamyl transferase
